# Experiences of rural clinicians accessing specialist support via telehealth for trauma and emergency care in Queensland, Australia

**DOI:** 10.1177/20552076241251950

**Published:** 2024-07-25

**Authors:** Chiara Santomauro, Mia McLanders, Andrew Rae

**Affiliations:** 1 Safety Science Innovation Lab, 5723 Griffith University, Brisbane, QLD, Australia; 2 School of Psychology, 1974 The University of Queensland, Brisbane, QLD, Australia; 3 Clinical Skills Development Service, Metro North Health, Brisbane, QLD, Australia

**Keywords:** Critical care, emergency medicine, rural and remote, telehealth, telemedicine, trauma

## Abstract

**Objective:**

Trauma and emergency patients presenting to rural facilities require time-critical treatment and management that is sometimes beyond the scope of clinicians in the facility. In Queensland, Australia's second largest state, telehealth infrastructure facilitates 24/7 communication between rural clinicians and tertiary-based critical care specialists. We sought to understand the current state of Queensland's emergency telehealth system from the perspective of direct end-users to inform future improvement efforts and resource allocation.

**Methods:**

Semi-structured interviews were conducted with 11 rural Queensland clinicians who use telehealth to access specialist support during critical presentations. Qualitative data were analysed in three inductive phases: immersion; a combination of process coding and in vivo coding; and focused coding.

**Results:**

The findings highlight that emergency telehealth support provides benefits beyond better patient care, as it fosters collegiality and alleviates professional isolation. Three key themes were identified: (a) strategies for overcoming challenges in providing trauma and emergency care in rural Queensland; (b) factors that affect perceptions of telehealth effectiveness; and (c) how support for rural trauma and emergency care can be improved. To provide context for the themes, a summary of scene-setting data is also provided.

**Conclusions:**

There are both advantages and disadvantages for rural clinicians accessing telehealth specialist support for critical care. Although telehealth is seen as a vital service that supports rural clinicians and benefits patient care, the findings suggest that tools, systems and processes surrounding rural trauma and emergency care could benefit from streamlining, integration, and the introduction of fit-for-purpose technologies. Addressing limitations of efficiencies would improve support for rural clinicians and likely improve patient outcomes for rural communities.

## Background

Almost one-third of Australians live outside major cities, with recent population growth in rural and remote areas caused by the COVID-19 pandemic and decentralisation of the workforce.^1,2^ Australians living in rural and remote areas are up to three times more likely to die a potentially avoidable death than those living in major cities.^3^ In locations like Queensland – Australia's second largest state with 5 million residents spanning over 1.7 million square kilometres – it can be challenging to overcome vast geographical barriers to provide equitable, high-quality healthcare to all patients across the state. Over the past few decades, telehealth has been revolutionary in reducing the barriers to healthcare access outside metropolitan areas.

Telehealth is the delivery of healthcare from a distance, enabled by information and communication technologies. The COVID-19 pandemic has highlighted the importance and benefits of telehealth in both rural and metropolitan areas.^4,5^ For people living rurally, telehealth improves access to specialist services and reduces the associated burdens (costs, time, inconveniences) of physically travelling to a specialised facility.^6^ Additionally, clinician-to-clinician telehealth benefits rural health practitioners through improved access to continuing education, collaboration and networking, and an improved ability to provide better local service.^6^

Trauma and emergency patients presenting to rural facilities require time-critical treatment and management that is sometimes beyond the scope and experience of clinicians in the rural facility. In most cases, these patients are aeromedically transferred to metropolitan hospitals for ongoing care, but it can take hours for the retrieval team to reach the rural facility and for the patient to reach definitive care. In response to this, the Queensland telehealth infrastructure facilitates 24/7 communication between rural clinicians and metropolitan tertiary- and quaternary-based critical care specialists who operate under the governance of Retrieval Services Queensland (RSQ).^7,8^ Over 110 rural emergency bays across the state are fitted with bidirectional videoconferencing technology (including ceiling-mounted cameras with remotely-controlled pan tilt zoom functionality, microphones, and television screens). This technology is used by critical care specialists (based in two of Queensland's largest cities) to remotely provide clinical advice to treating clinicians, such as decision support or procedural guidance, and to coordinate aeromedical retrievals to tertiary facilities for ongoing care.^9^

This model of care overcomes geographical barriers by making specialised expertise more accessible in rural facilities in time-critical situations. Even early on in implementation, the use of this telehealth infrastructure improved decision-making during the medical coordination of aeromedical retrievals across Queensland.^10^ Similar models of care exist globally, and consistently demonstrate positive outcomes for patients and clinicians.^11^ For example, an evaluation of an early teletrauma program revealed that the virtual presence of a trauma specialist changed treatment for 83% of trauma patients who presented to rural facilities.^12^ In a pre-post evaluation, the availability of a trauma specialist via videoconference significantly improved the care and management of rural trauma patients, including a dramatic reduction in the rate of patient transfers from 100% to 11%.^13^

Over the coming years, innovative technologies and tools will be introduced to advance the existing telehealth system.^7^ However, it is important to understand the current state of the system from the perspective of direct end-users to inform future improvement efforts and resource allocation.^14^ Therefore, we sought to explore the perceptions of Queensland's emergency telehealth system from the perspective of rural clinicians who use it to access specialist support. This study was conducted as part of a program of research designed to improve trauma and emergency care in rural Queensland by exploring the strengths and limitations of remote support methods and investigating novel communication technologies.

## Methods

### Participants and recruitment

Participants were drawn from a state-wide convenience sample of Queensland rural clinicians who use telehealth to access specialist support when delivering trauma and emergency care. Recruitment was conducted via email advertisements circulated through internal channels. A total of 11 participants were recruited on a volunteer basis. The number of participants was based on achieving geographic and role diversity, and aligns with guidance on the number of interviews necessary to achieve saturation in narrow-scope qualitative research.^15,16^ One participant was excluded because they did not use the emergency telehealth system, leaving 10 participants included in the analysis. Participant demographics are presented in Table 1.

**Table 1.** Participant demographics.

Demographic	Distribution (*N *= 10)
Gender	Female (8)
	Male (2)
Role	Director of Nursing (5)
	Rural Generalist/Senior Medical Officer (1)
	Nurse Practitioner (1)
	Clinical Nurse Consultant (1)
	Nurse Unit Manager (1)
	Clinical Nurse (1)
Queensland Hospital and Health Service (Note: the Queensland health system is divided into 16 hospitals and health services based on location)	Mackay (2)
	West Moreton (2)
	Cairns and Hinterland (1)
	Central West (1)
	Darling Downs (1)
	Metro South (1)
	Torres and Cape (1)
	Wide Bay (1)

### Procedure

One-on-one semi-structured interviews were conducted by author CS, who was not known to the participants prior, and took place online via Microsoft Teams (*n *= 9) and via phone call (*n *= 1). Interviews were held between March and August 2022 and lasted approximately 40–65 min each. Interviews were audio-recorded for transcribing purposes.

Participants were first asked to describe their general experiences using telehealth for specialist support during trauma and emergency care. Subsequent questions were inspired by the Critical Decision Method (CDM),^17^ which is particularly useful for analysing judgement and decision-making during tasks that are time-pressured, complex and dynamic, and identifying strengths and weaknesses in a process. In line with CDM, participants were asked to recall memorable experiences where they received specialist guidance through telehealth while treating a critically ill patient. They were then asked to recall specific details of the event using probes inspired by CDM. However, participants sometimes answered these questions more generally. The interviews concluded with general questions about challenges faced by rural clinicians and desired interventions to improve trauma and emergency support. Interview questions varied slightly depending on each participant's responses.

### Data analysis

Each interview was manually transcribed verbatim by a transcription service (Pacific Transcription). Transcripts were imported into QSR NVivo v1.6.1 software for coding. Since there were no a priori predictions of what themes might emerge, no underlying theoretical framework was used. Instead, this was an inductive approach to the generation of themes which emerged directly from the data. Data were analysed in three inductive phases: (a) immersion; (b) a combination of process coding and in vivo coding; and (c) focused coding. In the immersion phase, an in-depth review of each transcript was conducted to explore trends and themes that recurred throughout the interviews. In the first cycle of coding, process coding was adopted to denote both observable and conceptual actions in the data, by identifying and applying verbs to participant actions. This was paired with in vivo coding to capture notable language used by participants themselves.^18^ In the second cycle of coding, focused coding was used to identify the most significant categories in the data through an iterative process that involved inductively synthesising concrete verbs into more abstract actions to group codes together which share overarching themes.^18^ Author CS led the analysis with support from author AR in phase 3. To increase credibility of the results, the final step involved member checking by requesting and incorporating feedback from participants on categories and themes.^19^

### Researcher characteristics

Our research team consists of three PhD researchers. CS and MM specialise in Human Factors, concerned with the interaction between people and systems. CS is a research fellow who collaborates with healthcare organisations and MM is a healthcare-embedded researcher. AR is an associate professor in Safety Science with extensive expertise in qualitative methods.

### Ethics approval

This project was deemed exempt from full research ethics board review by the Royal Brisbane and Women's Hospital Human Research Ethics Committee (EX/2021/QRBW/79096) and was ratified at Griffith University. Prospective participants were given an information sheet and provided written informed consent prior to scheduling the interview.

## Results

Saturation was achieved after eight interviews, with the last two interviews generating no significantly novel insights. Three key themes were identified, each with two to three sub-themes, presented in Table 2. Quotations are presented with each participant's identification number in square brackets [1–10]. Apart from the state capital (Brisbane), town names have been deidentified. Participants sometimes referred to specialists as ‘RSQ’ or ‘telehealth’.

**Table 2.** Themes and sub-themes identified from interviews with rural clinicians about rural trauma and emergency telehealth support.

1	Strategies for overcoming challenges in providing trauma and emergency care in rural Queensland
1.1	Role shifting
1.2	Coordinating patient retrievals
1.3	Using telehealth to access support from specialists
2	Factors that affect perceptions of telehealth effectiveness
2.1	Perceptions of effective telehealth support
2.2	Perceptions of suboptimal telehealth support
3	How support for rural trauma and emergency care can be improved
3.1	Improving the way rural clinicians connect and communicate with specialists
3.2	Improving the way information is shared between rural clinicians and specialists
3.3	Reducing the burden on rural facilities

### Setting the scene

Queensland rural facilities are typically separated by large distances from their closest tertiary facility. Therefore, the majority of trauma and emergency patients are transferred to tertiary and quaternary referral centres via rotary- or fixed-wing aircrafts. Rural clinicians are physically isolated from their peers and feel the weight of this isolation when making clinical decisions. Physical distance also creates a barrier for education and training. Despite a hunger and appetite for education, some participants report that education is not prioritised because rural facilities struggle to cope when staff are away for training.

Rural facilities operate with fewer staff and resources than metropolitan facilities. Many rural nurses manage all patients alone or with one other nurse, and therefore work under extra time pressure to manage simultaneous competing priorities. Some facilities operate without a doctor, or with only a single doctor who may be on-call, but offsite. Some participants report that their facility is adequately staffed, but varying clinical experience and skillsets contribute to the challenging nature of rural practice.

Most participants acknowledged the difficulty in attracting and retaining rural staff. One participant mentioned that they only moved to their rural location because of a promotion and would otherwise not have. There is also concern for the changing workforce, with older clinicians retiring and being replaced with significantly less-experienced clinicians.

Rural clinicians live and work in towns with small, tight-knit populations, and are likely to see patients and family out in the community. Several participants recalled scenarios where the patient was known to them, making the events more meaningful and memorable. In these situations, participants are aware that their ability to be objective and impartial may be compromised. The death of a community member can be extremely traumatic and distressing for rural clinicians.

Rural facilities have limited labour and non-labour resourcing, depending on their size and location. Not all rural facilities stock every kind of medication or piece of equipment that may be required to treat and manage traumatic injuries. Pre-hospital resources such as ambulance vehicles are also scarce and must be carefully managed. Some participants report that their facility is ‘left behind’ when it comes to technologies. Many rural facilities are still paper-based and are awaiting technologies such as WiFi and videoconferencing equipment to be installed in their facility.

## Strategies for overcoming challenges in providing trauma and emergency care in rural Queensland

### Role shifting

One of the ways to deal with minimal staffing is role shifting, where staff perform tasks that are outside their usual scope of work. For example, rural nurses may perform tasks that would typically be performed by other specialties, such as doctors or paramedics. Some rural towns have no paramedics so the ambulance is driven by a volunteer community member and nurses are responsible for travelling to scenes. In areas with paramedics, they sometimes stay and assist in the resuscitation bay. Operational (non-clinical) staff also lend support, for example, by collecting equipment, moving patients and performing chest compressions. Rural clinicians have collegial relationships with other local staff and appreciate when they go beyond their role description to help during critical situations.

They’re normally the maintenance, the cleaners, they’re sort of that support person. But to be honest, they become quite multiskilled when working in these isolated areas like this…They know the equipment names and they know if you say ‘go and get that’, they’ll know where to go and get it. [3]

Community members, police, and patient relatives are also called on when additional hands are needed, although leading people who are not clinically trained can place additional stress on the clinician, especially when outside their own comfort zone (e.g., on scene).

We had to try and resuscitate them [on scene] and I didn’t have anyone there. It was me and one other nurse and some community members. So, I sort of team led…When you’re having community members or the police or someone else doing CPR and stuff, it can be difficult to make sure that your times are right. [5]

### Coordinating patient retrievals

Most rural trauma and emergency patients are transferred to tertiary and quaternary facilities for emergent treatment and ongoing care. Aeromedical retrievals are arranged through RSQ. To initiate a patient retrieval, rural clinicians contact RSQ via phone, who then arrange an aircraft and retrieval team to collect the patient and fly them to a larger hospital. Because rural facilities are typically not equipped to manage high-acuity patients, arranging an aeromedical retrieval is a top priority.

The workload required to facilitate the coordination of patient retrievals was inconsistent across participants and scenarios. Some participants reported that RSQ took care of all steps involved in the process, while others were frustrated with many time-consuming tasks needed to coordinate the transfer. ‘Finding a bed’ was one of the main burdens placed on rural clinicians who often described calling several different hospitals to find space for their patient before RSQ agreed to send an aircraft.

You’ve got to tell the [RSQ] nurse first and then she’ll put you on to the coordinator and then you’ve got to – usually she’ll talk to the coordinator, but then you’ve got to go through it again a second time…Then you’ve also got to tell the story a third time to the accepting hospital. So, it is minimum 20 min on the phone. Then on top of that you’ve got to then do your transfer paperwork. [1]

I think the thing for the doctors is that when you’ve made that call to Retrieval about getting a patient retrieved you have to also ring the accepting facility, have that conversation with the doctor in ED or if they’re going to be a direct ward admit…Sometimes Retrieval won’t come and pick a patient up if there is no bed. [9]

When RSQ handles most of the retrieval coordination, rural clinicians are very grateful.

Simultaneously they were sending someone to come and get him, which is fantastic. I can’t tell you how much that helps us on the floor. When we’re the ones that have to coordinate the transfer of a patient out of our hospital it can just be such a burden. [1]

[RSQ] booked all the transfers and everything for us, all that stuff that was excellent…It's the little things like not having to walk away from this to go and make a phone call to call a chopper. [2]

### Using telehealth to access support from specialists

One of the main strategies for managing critically ill patients is to connect with a critical care specialist via telehealth. This service is governed by RSQ so that aeromedical retrievals can be arranged simultaneously. Rural clinicians typically initiate a video call with a RSQ specialist when faced with time-critical, emergency situations, when they need specialised expertise, and when the level of knowledge and skill required is beyond that of the local team. Nurses working in facilities without doctors tend to rely more heavily on the service.

Rural clinicians employ several strategies to facilitate an effective telehealth experience. When there are several local clinicians treating a patient, a common strategy is to assign a local lead to converse with the specialist to minimise confusion or miscommunication.

I often find with the telehealth there’ll be sort of one lead [in the rural facility] and maybe there's 10 things going on behind the scenes, but at least the lead will be able to talk directly to telehealth and then guide the team. [5]

When the scenario becomes noisy, or when the patient's family are in the room and sensitive content needs to be raised, the local lead may mute the videoconference and call RSQ by phone.

We then muted both ends of the telehealth, and the lead doctor talked to the lead doctor in Brisbane by phone. So Brisbane could see everything that we were doing…It also allowed [RSQ] to say, ‘hey, have you considered that that might be a shaken baby?’ Whereas they couldn’t really say that over [telehealth] with the mother sitting there and the father sitting there. [6]

Rural clinicians also ensure that they are setting clear expectations and communicating their needs explicitly over telehealth to prevent delays or misunderstandings.

I think that initial communication about what the doctor on the floor actually expects from the RSQ and why that phone call has been initiated is really important in those beginning stages. [10]

## Factors that affect perceptions of telehealth effectiveness

### Perceptions of effective telehealth support

Specialist telehealth support can serve different functions in a critical situation depending on the patient's condition and local staff and resources available. Several participants recalled situations where the specialist adopted a team leader role and watched over the entire situation, which tends to happen most when there is no local doctor on site or not enough clinicians to assign a local team leader.

When I’m the only one that can do all the different procedures, it's really hard for me to maintain that helicopter view of the whole resuscitation. So, I really like to have somebody who's literally looking over my shoulder to help direct and guide and make sure that things aren’t being forgotten. [1]

So I actually feel like, if you can say, but like an angel in the sky watching down sometimes is, yeah, it's actually just another set of eyes…RSQ is amazing because they’re watching what the clinical lead at the end of the bed should have been. [10]

Consistent with a team leader role, and because they are not preoccupied with their own manual tasks and have a whole-room view, specialists support the local team by keeping situation awareness of the entire scenario and providing timely input.

[RSQ] had the eyesight to be able to see ‘okay, this is deteriorated. He needs to be intubated. You need to do this.’ Because sometimes, as you would know, there's so much going on in that room that we do need that voice from above. [2]

Specialists also provide rural clinicians with someone to problem solve and make joint decisions with. Their support increases confidence in decision-making and clinical management.

I think that's the power for a doctor as well is having another senior head to bounce things off because they’re quite literally on their own…Sometimes it's just that conversation that a doctor has with another doctor to trouble[shoot] – to brainstorm, really. [8]

Other times, the specialists provide more specific direction, guiding rural clinicians step-by-step through tasks such as medication preparation and administration. This is particularly helpful when managing a new, complex, or highly time-critical scenario. In such cases, specialists share the mental load by taking over tasks that can be done remotely, such as scribing and calculating drug doses.

They’ll document our drugs and everything for us. They’ll send the email or a fax or send you the medication chart because in those really small facilities like [rural town], a scribe's a luxury. We don’t have those. [2]

It was easier for the consultants to say, ‘right, now you need to do 1 in 1000 then draw it up to 10 millilitres and then do it again’. So, that made it a lot easier from that part, and probably a lot quicker and more timely. [7]

Some participants mentioned that when the specialist's communication style involves approaching scenarios with curiosity rather than authority, this helps to foster a safe and supportive environment.

I always like the fact that they say things like ‘have you considered this’ as opposed to ‘you should do this’. [2]

In addition to providing clinical guidance, specialists also assist with other aspects of care such as managing the emotional wellbeing of the patient's family. A specialist's virtual presence alone can provide reassurance to families that the local team is doing everything they can. Specialists can also speak directly to families to provide impartial assurance about the local team's decisions and actions, or in some cases, help the family make difficult decisions.

We were resuscitating a child that was quite dead, we really needed the RSQ to have that conversation with the parents. So I suppose it is a little bit like we wanted them to support us, but it was that they were helping the parents make that decision [to stop resuscitation]. [6]

I know it sounds really silly but having that metropolitan support too helps with the community because they feel like you’ve done literally everything you possibly can for that patient. [10]

Rural clinicians report that the visual information displayed in a videoconference impacts specialists’ decisions and directions and benefits patient care more than a phone call. Some participants also mentioned that the local context is better understood during video calls which enhances collaboration between the two teams.

You can explain what something looks like in front of you, but until people see it, you don’t really – it's hard to put the whole picture together. So maybe we would have had different advice surrounding her burns and her dressings and the cooling process, so – does that mean potentially she would have ended up with compartment syndrome? [5]

We were busy trying to do stuff and it was again that situational awareness and the telehealth doctor just said, ‘oh, that's stertorous breathing’…We just went, ‘oh, crap. His head injury is a lot further down the line than we thought’. [8]

In some scenarios, participants reported that outcomes could have been catastrophic for the patient or rural team if no telehealth support was available.

So, I think potentially things could’ve been very different. The child might have died. [9]

I think the team would have broken. [10]

All participants reported that emergency telehealth support provides an additional layer of protection to rural clinicians and helps them feel safe, supported, confident and less alone in their practice.

[RSQ] never left us alone for 12 hours which was really, really reassuring and impressive for all of us. [5]

With RSQ, I’ve always got a sense of relief when they’re there, like a safety net. [9]

### Perceptions of suboptimal telehealth support

While recognising telehealth's critical role in rural trauma and emergency care, participants described some common issues that can make the experience more effortful and less efficient. Many participants recalled situations when the specialist made incorrect assumptions about the rural facility's resources, which can lead to misalignment between the specialist's directions and what is practically possible. However, participants acknowledged that because of the large variability in Queensland facilities, it is very difficult for specialists to know or retain this information, and the team is usually able to devise alternative plans and work around limitations.

They may not know the capability of the facility. So whether there's pathology on site, or whether you can do an X-ray, and I think it would be very hard for them to understand that for every facility across the state. [6]

Sometimes they tell you to do lots of stuff, and I think it's because they’re used to being in a big ED where you’ve got five or six people to be able to do things and they’re saying, ‘right, do this, draw this up, do that’, and you’re like, ‘I’m one person’ [laughs]. [7]

Specialists also may not be aware that rural clinicians are managing stressful or tense situations outside the resuscitation bay. These situations can influence patient management decisions but may not be fully understood by the specialist.

Sometimes I don’t think they understand how volatile our workplace can be…[Specialists will] be like, ‘we need to stop’. Yes, we know that we need to stop, but we also know that we’ve got maybe 100 people outside who are very emotional, sometimes intoxicated. So, I think sometimes they don’t understand that maybe if we just stop it's going to become potentially really dangerous. [5]

RSQ can only see what's happening on the bed, they can’t actually hear the commotion going on outside…It can escalate sometimes where the family members may be banging on the doors at the same time as, you know, you’re trying to do things…you’re trying to manage that situation as well as what's going on outside, and RSQ are just focused on getting the patient [stable]. [7]

Some participants expressed frustration around the process to engage RSQ for telehealth and retrievals. The process was described inconsistently across participants and may vary depending on the Queensland health service or staff involved. Some participants described the process as time-consuming whereas others recalled situations where videoconferences were initiated instantly and effortlessly. Sometimes rural clinicians must wait for a specialist due to competing demands across the state.

There's been times where we haven’t been able to get hold of RSQ straight away and it's been quite a lengthy process and that time can feel like forever. [10]

If there are delays connecting with RSQ, the specialist may join the videoconference well into the scenario.

Because we hadn’t dialled in super early…There was a lot of backtracking for them to catch up with what had been going on…But it felt frustrating at the time because it was that essence of – time is of the essence. [8]

Sometimes rural clinicians have trouble hearing specialists over telehealth. This typically happens when other specialists (e.g., paediatric, neurology, burns) join the videoconference to provide further expertise, or when there are lots of people in the rural resuscitation bay (e.g., when paramedics assist the rural team). This can also make it difficult to maintain control of the situation.

There were actually so many [ambulance] staff there and so much talking and noise that I lost communication with the retrieval doctor. So, I remember phoning him up afterwards and he was saying he was yelling to try and get my attention and the attention of my team and they just couldn’t. [1]

I think it may have been a paediatric consultant as well as the RSQ consultant…And because it cuts out, you sort of miss half of what the other one was saying. [7]

Although rare, there were some recollections of conflict between rural clinicians and specialists.

I’ve seen doctors hang up on RSQ and just be, like, ‘oh, that's it, see you’ [laughs]. Which adds to the tenseness of the moment…And the cameras go off and you’re, like, ‘oh, crikey’ [laughs]. [10]

Rural facilities experience more frequent power outages which impacts the quality of support RSQ can provide and in some cases can mean that no communication with RSQ is possible.

It might be two to four hours – our phone lines go down. So then obviously we lose that form of communication. [5]

We’d often get brownouts out there with the power, so that would turn the [telehealth] system off. [9]

The process of exchanging information with RSQ was another inconsistency across participants. Some described using email to exchange information but others expressed frustration with outdated technologies still being the standard method to transmit information.

I still fax ECGs through to RSQ, like, 100 per cent fax. So, they’ll give me the fax number, I’ll have to run out of the department to the reception, fax them, hope to God I’ve given the right number, run back. I’m just like, ‘this is 2022’. [1]

Normally it's faxing, which I don’t understand in this day and age why we still fax? It's insane. You may as well send it by pigeon, or something. It's ridiculous. [9]

Sending patient images to RSQ digitally can be useful but doing so has inherent privacy concerns and there does not seem to be a formalised, secure process to send images. Participants use workarounds, but the most secure workarounds are cumbersome and the easiest workarounds carry higher risk of breaching patient confidentiality.

A lot of people take photos with their phone and text them, but that's completely inappropriate, like, you can breach confidentiality – I don’t do that. We do have a clinical iPad that we can use to take photos and email them through the Queensland Health network, so that's one way around it. But that still takes time. [1]

We often take photos of things and text them to the retrieval doctor so that – well, that's something that I’ve noticed, anyway, I don’t know if it happens in every resus but I notice it happening so maybe that's happening informally. [8]

## How support for rural trauma and emergency care can be improved

### Improving the way rural clinicians connect and communicate with specialists

Rural clinicians report feeling frustrated about the amount of time they spend on the phone to RSQ and tertiary facilities to initiate videoconferences and arrange patient transfers. Several participants want portable solutions such as wireless headsets so they can perform manual tasks while on the phone. This could also benefit those situations where the local lead speaks to the RSQ specialist via phone during a videoconference.

It just comes back to time on the phone, so a headset would be amazing because then I could put the cannula in or just look after my patient hands-free while I talk. [1]

Even with mobiles, it's still like – you’re trying to do stuff and then hold a phone and talk as well. So, something that you can do hands-free and be able to be attending to the patient and be communicating to the support at the same time, is certainly a lot more advantageous. [3]

Rural clinicians also want a simplified process for contacting services such as RSQ, especially for those who work alone or with minimal staffing. Suggestions centred around a switchboard-type system that would allow direct contact with specific departments, or even specific specialists, at the click of a button.

Because we transfer to [city]…Often those specialists already know our patients and we’ve got such complex patients, I could just press their name on whatever thing and they’d come up…And then just trying to explain people's past medical history and while they’re really, really unwell, but you absolutely have to. You’re trying to do 10 things at once and you’re like, this is really hard. [5]

When you’re on your own or when there's only two of you there, to be able to get through a lot quicker than actually having to phone through…Even if there was a way of doing it where you just hit one button on the iPad or something. [7]

Another recurring suggestion was to improve portability to allow communication with specialists while working in prehospital environments, which could be especially useful in rural communities with no paramedics (where, for example, a nurse may travel to scene).

I’d like a GoPro and telemetry with my lifepack in my ambulance to be able to talk to [RSQ]. [4]

If I’m going out to [scene]…I guess I’d just be like, ‘can I facetime you?’ I know that's not what we’re supposed to do, but I need some help…It would be very handy to have an option that you can take even outside of your place of work. [5]

### Improving the way information is shared between rural clinicians and specialists

There were several discussions around information sharing systems to allow rural clinicians to send, and specialists to access, information more easily. Suggestions like interactive whiteboards that would allow both parties to share information (e.g., medications, imaging) on a collaborative virtual platform, and software that allows high-resolution images to be shared seamlessly and instantly, were put forward.

If they’ve got a compound fracture I could just take a photo of it and then the ECGs and that could just automatically be on some shared platform where they can see it without me having to leave and go to a fax machine or leave and email it, that would be amazing. [1]

Several participants discussed the lack of integration between systems. Information is documented in different systems that not everyone has access to, which means that sharing information across different sites can be effortful, inefficient, and time-consuming. Therefore, participants expressed desire for everything to be consolidated and stored in a single system that specialists can access to reduce the workload of rural clinicians. Some participants report that digital health records, which are not currently implemented in all facilities, will improve integration of patient information across facilities.

We need a system where the guy at telehealth can just go, we can just shift that information to them or they can log in or something…I know that that's probably headed that way with electronic chart but I also know from the rural perspective, we’re a hell of a long way from that happening. [2]

The concept of interactive telepresence is being investigated by the research team as part of the overarching project so the concept was presented to all participants to gauge initial opinions. Interactive telepresence is designed to facilitate physical interaction by allowing the specialist to guide the rural clinician through an intervention by virtually annotating on the patient in real time, for example, through augmented reality on a head-worn device.^20^ Impressions were positive, with some participants providing practical considerations.

You are talking them through doing these procedures. Why not show? Show often is better. I think it would be really good with some wounds, too. [2]

I think for me something that would go up onto the big screen that would live on the wall [would be better]. Yeah just from a pure – you’re trying to do three things when you’re only one person. So you’re trying to put the IV in when taking the blood pressure. So yeah having something that's just there in front of you, not that you’ve got to go look for and then think, ‘where the hell did I put the iPad’. [4]

### Reducing the burden on rural facilities

Rural practice inevitably involves multitasking, but a recurring frustration was the burden of tasks placed on rural clinicians despite being under-resourced and under-staffed. The responsibility of several tasks could be shifted to other services (e.g., RSQ) to reduce workload during trauma and emergency care, such as finding an available tertiary bed for the patient.

If they could take away the coordination so that I didn’t have to phone up three or four different hospitals for one case, that would be amazing. [1]

If you just had to make one phone call, and things just magically happened, they came and picked the patient up, and they decided where they were taking the patient, happy days. It would take the onus off the rural facility and then the staff in the rural site actually could focus more on the care of that patient. [9]

Another task that could be shifted to RSQ is scribing during critical scenarios because it can be done remotely. Currently, this occurs in only some cases and likely depends on factors such as RSQ nurse availability at the coordination hub, or the number of rural staff available. Several rural clinicians have never had RSQ scribe for them but want this to happen.

A team of three is a little bit hard and sometimes next to ‘scribe’ they write ‘telehealth’…[But] I just don’t think that's something that they do. But in a little place if it was something they could do, damn, it would be good…At the moment what we do is we just keep every single drug vial, we chuck it on the bench and at the end we’re like raking through them, making sure we wrote them all down on the med chart. [8]

Several participants stated that they simply need more staff in their facilities. Others mentioned that they need more skilled and experienced staff and consistent doctors for their community. To improve skills and experience, rural clinicians want better access to education and training, with more standardised training across the state. Maintaining skills and upskilling is challenging for those located far from larger cities. With rural practice being so broad, one participant suggested a rural generalist pathway for nurses, similar to what is available for rural doctors. To make training more accessible, another participant suggested that simulation training could be delivered remotely by educators via telehealth which would remove the need for travel and associated inconveniences.

If you’ve got the right people in the building, it doesn’t matter what the building's like, you can give good care. [6]

## Discussion

In rural areas, it can be challenging to overcome vast geographical barriers to provide equitable, high-quality healthcare to all patients. The purpose of this study was to investigate clinicians’ perceptions of trauma and emergency telehealth support in rural Queensland. Three key themes emerged from the data: (a) strategies for overcoming challenges in providing trauma and emergency care in rural Queensland; (b) factors that affect perceptions of telehealth effectiveness; and (c) how support for rural trauma and emergency care can be improved.

Consistent with prior literature,^21^ our sample of rural clinicians typically work in resource-poor facilities and experience feelings of isolation as a consequence of geographical distance. Rural clinicians employ several strategies to compensate for challenges specific to rural trauma and emergency practice. For example, rural clinicians are frequently required to work outside their typical scope of practice,^22^ and our data revealed that role shifting is used to compensate for skill mix defecits to ensure that what needs to be done gets done. Another strategy is transferring patients to better-resourced tertiary facilities as soon as possible^9^; however, in our sample some clinicians revealed that they experience a burden of responsibility in contributing to the coordination of patient retrievals, whereas others experienced full support of coordination by telehealth specialists. A consistent approach for the management of patient retrievals across all rural sites would improve planning and workload management.

To support trauma and emergency management at the rural facility, critical care specialists provide crucial guidance to rural clinicians via telehealth, which is a successful model of care employed in other areas around the world.^11,23^ The findings from the present study revealed several benefits of telehealth. Telehealth can mitigate the challenges of isolation and skill mix deficit by allowing a remote specialist to virtually ‘step in’ as the team leader and use their bird’s-eye view to maintain awareness of the entire situation. Effective remote support can also look like supporting decision-making, providing step-by-step direction, and sharing the mental load. An unexpected finding was that specialists may also assist in supporting the emotional wellbeing of the patient's family, which is particularly important in cases where the patient is known to the treating clinician (as is common in rural communities^24,25^). Most importantly, telehealth changes the trajectory of care by providing real-time visual feedback and access to a broader range of expertise. As a consequence, rural clinicians feel highly supported when using telehealth.

Despite the advantages, the data also revealed disadvantages of telehealth that made accessing support more effortful and less efficient. Rural clinicians reported that specialists do not always understand their facility's limit of resources, or the demands and intensity of rural practice. In some cases, having more people involved in the care was not necessarily better and led to difficulties controlling the situation and hearing specialists over telehealth. In the first instance, specialists with clinical experience in rural facilities will be better placed to provide the right kind of support, and in the second instance, strategies such as structured communication protocols may improve communication challenges.

Another barrier was technological issues, such as power outages, which are common in rural areas. Further, digital exchange of information was frustrating in facilities where outdated technologies such as fax machines were still in use. More investment in suitable infrastructure requires a commitment from government and telecommunication companies beyond current political and commercial priorities.

Finally, several ways that support for rural trauma and emergency care could be improved emerged from the data, ranging from simple interventions to entire overhauls of systems. For example, rural clinicians want more efficient and simplified processes to connect and communicate with specialists to reduce time spent on the phone, and requested headsets to maximise efficiency while on calls. Improving the portability of telehealth would allow support in prehospital environments. However, there was some apprehension towards portable devices because they can be misplaced or stolen.

Rural clinicians want to exchange information with specialists more easily. Novel technologies such as collaborative virtual platforms, systems that integrate information from several other systems, and augmented reality devices may offer solutions to improve digital information exchange. However, factors contributing to the success and adoption of healthcare innovations should be considered when implementing such technologies in rural facilities.^26^ The Queensland telehealth system is currently undergoing an array of changes as technology advances and infrastructure is upgraded,^7^ and some of the improvements suggested in these interviews are already under investigation.

Trauma and emergency support could be improved by reducing the burden currently placed on rural facilities. The responsibility of some high-workload tasks could be redirected elsewhere, such as scribing for a resuscitation or locating an available bed for a patient transfer. Employing more skilled staff and improving access to education and training (e.g., remotely delivered through telehealth) would place rural clinicians in a better position to deliver consistent, high-quality care to critically ill patients. Despite statewide support from the Clinical Skills Development Service, training and education opportunities are difficult to access in these areas, in part due to limited staff resourcing to enable professional development leave. This points to the need for a dedicated task force to ensure that training needs are consistently identified for rural sites and implemented at a state-wide level.

Due to the size of Queensland, the health system is divided by location into 16 hospital and health services. The findings are limited to a convenience sample of 10 rural clinicians who came from 8 of the hospital and health services, so it is possible that other themes may have emerged across different geographical locations. Additionally, Queensland's telehealth and retrieval system is unique, so not all findings may generalise to other states or countries. Future research could investigate which themes are shared at a national or international level. Generally, participants provided relatively balanced views (i.e., positives and negatives) of the telehealth and retrieval system; however, sampling bias could be present because the study may have attracted participants with a stronger interest in telehealth compared to the general rural clinician population. Finally, this study presents the perspectives of rural clinicians who represent only one half of the telehealth end-user population. Specialists who provide remote support via telehealth were interviewed in a related study. They provided unique perceptions so their data will be shared separately.

## Conclusions

The purpose of this study was to explore trauma and emergency telehealth support from the perspective of rural clinicians working in Queensland, Australia. Telehealth specialist support is a critical service that provides benefits beyond better patient care, as it fosters a sense of collegiality and alleviates professional isolation. However, the findings also revealed disadvantages of telehealth that made accessing support more effortful and less efficient. The findings suggest that tools, systems, and processes surrounding rural trauma and emergency care could benefit from streamlining, integration, and the introduction of new fit-for-purpose technologies. Maximising the efficiency of the emergency telehealth system would lead to better support for rural clinicians and ultimately improve patient outcomes for rural communities who are currently disadvantaged when accessing health services.

## Supplemental Material

sj-docx-1-dhj-10.1177_20552076241251950 - Supplemental material for Experiences of rural clinicians accessing specialist support via telehealth for trauma and emergency care in Queensland, Australia Supplemental material, sj-docx-1-dhj-10.1177_20552076241251950 for Experiences of rural clinicians accessing specialist support via telehealth for trauma and emergency care in Queensland, Australia by Chiara Santomauro, Mia McLanders and Andrew Rae in DIGITAL HEALTH

sj-docx-2-dhj-10.1177_20552076241251950 - Supplemental material for Experiences of rural clinicians accessing specialist support via telehealth for trauma and emergency care in Queensland, Australia Supplemental material, sj-docx-2-dhj-10.1177_20552076241251950 for Experiences of rural clinicians accessing specialist support via telehealth for trauma and emergency care in Queensland, Australia by Chiara Santomauro, Mia McLanders and Andrew Rae in DIGITAL HEALTH
