# Supplementary material for: Experiences of rural clinicians accessing specialist support via telehealth for trauma and emergency care in Queensland, Australia
Source: Digit Health. 2024 Jul 25;10:20552076241251950. doi: 10.1177/20552076241251950 (PMC11282562; doi:10.1177/20552076241251950)
Supplement: sj-docx-1-dhj-10.1177_20552076241251950 - Supplemental material for Experiences of rural clinicians accessing specialist support via telehealth for trauma and emergency care in Queensland, Australia [file sj-docx-1-dhj-10.1177_20552076241251950.docx]

**Experiences of rural clinicians accessing specialist support via telehealth for trauma and emergency care in Queensland, Australia**

**Interview Questions**

| **Interview Component** | **Question** |
| --- | --- |
| **Recall Questions (Inspired by CDM)** | Let’s begin by taking a few moments to describe your general experience with accessing remote support from specialists. |
|  | Can you now recall a memorable telehealth experience that you had, where you accessed support from a critical care specialist via telehealth while you were treating a trauma or emergency patient.   - Memorable event = one you can remember clearly, it doesn’t have to be nonroutine or difficult - Only interrupt with clarification questions |
|  | Why was this experience memorable for you? |
|  | Who was involved? (if not already recalled) |
|  | How would you describe the teamwork during this event? |
|  | What were the biggest concerns, difficulties, or limitations you experienced during this event with regards to telehealth? |
|  | What was your biggest frustration with regards to the technology? (if different from prior question) |
|  | How timely was the information that you needed being shared with you? |
|  | At any stage, were you uncertain about the reliability of the information that you had available? |
|  | What was the most important piece of information that you used to formulate your decisions? |
|  | Was there any additional information that you would have liked to share with the specialist during the event? |
|  | Was there any additional information that you would have liked to receive from the specialist during the event? |
|  | At any stage, did it seem as though the technology slowed things down or caused a distraction? |
|  | Were there any barriers to accessing support as a consequence of the technology? |
|  | Did you at any time feel like the decisions and actions you were making were constrained by standards/rules/procedures or the technology available to you? |
|  | How responsible did you feel for the events that occurred and the outcomes? |
|  | Did you feel like you had full situation awareness? Why/why not? |
|  | Did it seem like everyone involved had a shared understanding of the situation? Why/why not? |
|  | Does this event fit a standard or typical event that you’d be requesting support for? |
|  | What do you think would have happened to the patient if no remote support was available? |
|  | Could you have accessed support without telehealth? What would that have looked like? |
|  | Can you recall how you felt or what you were thinking about afterwards? Not only immediately after but in the hours that followed (for example, while you were travelling home) |
|  | How long did those thoughts and feelings stay with you? |
| **General Questions** | What do you think is the biggest challenge for rural clinicians? |
|  | If we were to design an intervention—it could be a training intervention, or a piece of equipment, or a system, or an extra personnel resource—to improve current remote support methods, what would it look like?   - Can think about this in the context of the recalled event |
|  | Would it have been helpful if you were able to receive visual guidance from the specialist on a portable device, in the form of annotated patient images in real time? For example, marking incision points or anatomical landmarks.   - Can think about this in the context of the recalled event |
| **Wrap Up** | Thanks very much for sharing your experiences with me. As I mentioned at the start, the reason why we’re doing these interviews is to understand the positives and negatives of the telehealth system and to develop some recommendations to improve it.  Before we wrap up, is there anything else that I should have asked or anything else that you want to say on this topic? Is there anything else that you thought of during the interview that you’d like to mention? |
